# Attitudes Towards Aging, Depression, Physical Functioning, and Pain Among Women Living with HIV of Reproductive Age

**DOI:** 10.1007/s10461-025-04724-9

**Published:** 2025-05-06

**Authors:** Emily M. Cherenack, Theodora F. Brophy, Margarita Avila Max, Alicia Graubard-Silebi, Nicholas F. Nogueira, Yue Pan, Daniel Westreich, Elizabeth F. Topper, Deborah Konkle-Parker, Aadia Rana, Seble G. Kassaye, Anandi N. Sheth, Deborah L. Jones, Maria L. Alcaide

**Affiliations:** 1 Department of Psychiatry and Behavioral Sciences, University of Miami Miller School of Medicine, Miami, USA; 2 Department of Medicine, University of Miami Miller School of Medicine, Miami, USA; 3 Department of Public Health Sciences, University of Miami Miller School of Medicine, Miami, USA; 4 Department of Epidemiology, University of North Carolina at Chapel Hill, Chapel Hill, USA; 5 Department of Epidemiology, John Hopkins University, Baltimore, USA; 6 Schools of Nursing, Medicine and Population Health, University of Mississippi Medical Center, Jackson, USA; 7 School of Medicine, University of Alabama at Birmingham, Birmingham, USA; 8 School of Medicine, Georgetown University, Washington D.C., USA; 9 Department of Medicine, Emory University School of Medicine, Atlanta, USA; 10 Department of Obstetrics and Gynecology, University of Miami Miller School of Medicine, Miami, USA; 11 Division of Infectious Diseases, Department of Medicine, University of Miami Miller School of Medicine, Miami, USA

**Keywords:** Aging, HIV, Women, Attitudes towards aging, Health-related quality of life, Depression

## Abstract

Positive attitudes towards aging (ATA) are associated with better mental and physical health in the general population and with lower depressive symptoms among men living with HIV. Little is known about ATA among women of reproductive age living with HIV (WWH), who often experience premature aging, depression, and chronic pain. This study examined the association of ATA with HIV, physical functioning, and pain among women 19–45 years-of-age. From 2021 to 2022, survey data were collected at baseline from 143 WWH and 39 women without HIV (WWOH) across the southern United States; 94 WWH also completed a six-month follow-up visit. Baseline ATA was compared across HIV status. Among WWH, correlations between ATA, participant factors relevant in prior research (i.e., age, depression, illness), pain, and physical functioning were examined. Regressions examined the association of baseline ATA with physical functioning and pain across visits, including when controlling for potential confounders. Having one or more negative ATA was reported by an equal proportion of WWH (57%) and WWOH (58%). Among WWH, more positive ATA correlated with lower depressive symptoms, better physical functioning, and lower pain at baseline. Lower depressive symptoms correlated with better physical functioning and lower pain at baseline. In regressions, more positive ATA was associated with better physical functioning at baseline and follow-up. A weak association of ATA with pain at baseline was not significant at follow-up or after controlling for depression. Research is warranted to examine how combined interventions to improve ATA, depression, and pain can support well-being among reproductive-aged WWH.

## Introduction

Although antiretroviral therapy has decreased AIDS-related deaths among women living with HIV (WWH) [1], WWH experience faster biological aging and a higher rate of co-morbidities at younger ages compared to women without HIV (WWOH) [2–4]. Per Flood’s [5] successful aging theory, successful aging involves the ability to cope with changes from physical and functional decline and to achieve overall life satisfaction with a sense of meaning in life, which is influenced by psychological factors, including perceptions of aging, and physical factors, such as chronic disease. The field of psychoneuroimmunology has begun to elucidate how psychological attitudes (e.g., optimism, purpose in life) and stress influence the immune system and drive aging processes through dual biological mechanisms (e.g., immune cell functioning, neurotransmitter activity) and behavioral mechanisms (e.g., isolation, reduction in physical activity) [6–8]. Consequently, biological and immune-related factors related to HIV and the natural aging process may interact with stigma, stereotypes, and attitudes about aging and HIV to influence well-being [9, 10]. For example, older adults with HIV report experiencing intersectional stigma as a result of co-occurring HIV stigma and ageism [11, 12], and HIV stigma among WWH has been shown to predict pain via an indirect path through depression [13]. Modifiable psychosocial factors, including self-perceived aging, mental health, and coping with aging, may serve as intervention targets to enhance well-being [9, 10].

Attitudes towards aging (ATA) include the self-stereotypes, expectations, attributions, and beliefs about how aging will impact one’s own well-being [14, 15]. Across populations of people without HIV, having a more positive ATA is associated with better health-related quality of life and physical and mental health [16–19]. In one large longitudinal cohort study of men and women over 50 years-of-age in the US, having above-average (i.e., more positive) ATA predicted 7.5 years longer median survival across 23 years of follow-up, as well as better functional health across 18 years of follow-up [17, 18]. The relationships of ATA with future mortality and functional health remained significant after controlling for covariates such as age, gender, socioeconomic status, and self-ratings of health [17, 18]. Among 6095 adults over 50 years-of-age in Ireland, more negative self-perceptions of aging at a baseline visit predicted the onset of new depression and anxiety, as well as the persistence of existing depression and anxiety, at a two-year follow-up visit [20]. In one study among married women over 50 in the US, ATA predicted self-rated health and depression measured eight years later [19]. Lastly, in a study of 5702 adults in the US over 60 who were not obese at baseline, having a positive ATA predicted a lower likelihood of becoming obese over the following six years after controlling for age [21]. Associations between ATA and physical functioning and mental health have also been confirmed in cross-sectional research and in global settings [22–24].

There are several cognitive, behavioral, and social mechanisms that could explain the bi-directional associations between ATA and health outcomes. More positive ATA have been associated with higher participation in health-promotion behaviors [25], including physical activity [23]. Among men and women 50–70 years-of-age in Germany, belonging to a latent cluster defined by “conventional” ATA, including believing that that “physical deterioration and dependence are a fact of life in old age,” and “withdrawing and keeping a low profile is appropriate behavior in old age,” was associated with lower likelihood of regular physical activity and healthy diet, even though individuals in this cluster often endorsed the importance of physical activity and diet [26]. In the longitudinal cohort of US adults, the associations between ATA and physical functioning and mortality were partially mediated by differences in perceived control (e.g., “I can do just about anything that I set my mind to”) and descriptions of life in retirement as more “full”, “hopeful”, and “worthy”, respectively [17, 18]. ATA have shown associations with perceived stress, depression, anxiety, and loneliness, which can all have negative impacts on health [16, 20, 22, 24, 27–30].

Less is known about ATA among people living with HIV (PWH). A cross-sectional study of 700 older adults in China found that ATA were more negative among PWH and were associated with having elevated depressive symptoms, which was also more common among PWH [31]. In contrast, a study among men over 50 years-of-age in the US in the Multicenter AIDS Cohort Study found no differences in ATA by HIV status, but did find that men across HIV status with depressive symptoms were more likely to have the most negative ATA [32]. No studies have been conducted on ATA and health-related quality of life over time among WWH in the US. While ATA appear to be modifiable via targeted cognitive-behavioral therapy, such interventions have not been tested among WWH [33].

Research is needed to examine psychosocial factors related to successful aging among women of reproductive age, a stage prior to mid-life during which negative aging experiences can begin to occur. Attitudes towards aging begin to form as early as 4–7 years old and become internalized in later life as attitudes towards one’s *own* aging [15, 34]. Research on college-aged women in the US has identified higher concerns about changes in physical appearance due to aging compared to men, with college-aged women predicting 48 is the age at which they would begin to feel “old” [35]. A qualitative study among younger adults (18–30) demonstrated that feelings about aging became more negative during the COVID-19 pandemic, especially with respect to concerns about age-related health risks [36]. However, studies on aging attitudes among women of reproductive age have tended to focus on concerns about future aging and not current ATA, despite the fact that many age-related concerns, such as worries about fertility, impact women in their 30s and 40s. Continuity theories of aging posit that—in the absence of intervention—coping strategies and psychological perspectives often remain consistent across age, which suggests that earlier ATA may predict future aging outcomes [37, 38]. There is currently a gap in research on ATA and health outcomes among reproductive-age women. The current study examined ATA among women 19-to-45 years old as the first step in the development of psychological interventions to enhance successful aging arising in the reproductive years.

Consequently, the current analysis aimed to answer the following questions: (a) Do ATA differ between WWH and WWOH, (b) are ATA associated with pain and physical functioning among WWH, and (c) do ATA among WWH remain associated with pain and physical functioning after controlling for theoretically selected covariates? It was hypothesized that more positive ATA would be associated with better pain and physical functioning at baseline and at a six-month follow-up visit, and that these associations would remain significant after adjusting for theoretically selected covariates.

## Methods

### Participants

This study includes a cross-sectional and longitudinal analysis of survey data collected from a subset of women who were enrolled in the Study of Treatment and Reproductive Outcomes (STAR) in 2021 and 2022 [39]. As of 2024, STAR is an ongoing multi-site longitudinal cohort study being implemented in the following six cities across the southern US: Atlanta, Georgia; Washington DC; Miami, Florida; Jackson, Mississippi; Birmingham, Alabama; and Chapel Hill, North Carolina. STAR is currently enrolling reproductive-age WWH and WWOH at heightened behavioral vulnerability to HIV in a 2:1 ratio. Inclusion criteria for all women in STAR included being between 18 and 45 years of age at screening, being a cisgender woman, and being willing and able to provide informed consent and complete study procedures in Spanish or English. Eligible WWOH were required to report at least one sexual behavior or substance use behavior indicating elevated HIV risk, or to have a sexual partner living with HIV or at high risk for acquiring HIV, in the past five years. HIV status was confirmed through documentation of positive HIV status or HIV 4th generation rapid antigen/antibody testing at screening. Recruitment varied across sites, but generally involved recruiting from the local clinic population, consent-to-contact databases, and community organizations and events.

The current analysis excluded (a) participants with incomplete data on ATA at baseline, which primarily excluded women enrolled after mid-2022 as the full ATA measure was not delivered, and (b) pregnant women due to physical effects of pregnancy. For analysis of follow-up data, only the subset of participants with complete follow-up data on physical activity and pain were included. A minimum sample size of *n* = 55 was calculated a priori using G*Power to detect medium-sized effects (f^2^ = 0.15) with a significance level of 0.05, power set to 0.8, and five predictors in a linear regression [40]. The size of the baseline (*n* = 143) and six-month follow-up samples (*n* = 93) of WWH are larger than the minimum sample size. The small sample of WWOH (*n* = 39) is one reason why WWOH were only included in the analyses comparing ATA across HIV status, for which the total sample size was *n* = 182.

### Procedures

Procedures were approved by the University of Miami single IRB (IRB protocol number 20190953). All participants completed a written informed consent process prior to enrollment and attended a baseline study visit to provide biospecimens and complete an interviewer-administered psychosocial survey, which included additional cohort study measures not included in the current analysis [39]. Participants were asked to complete a follow-up visit about six months after the baseline visit, which included assessments of physical functioning and pain.

## Measures

### Sociodemographic Variables

Sociodemographic variables at baseline included age, race, ethnicity, and difficulty paying for basic needs, which were used to characterize the sample.

### Attitudes Towards One’s Own Aging

Baseline ATA were measured using the Attitude Towards Own Aging Subscale of the Philadelphia Geriatric Center Morale Scale [41], which has previously been used in research among people living with HIV [32]. Participants were asked the following questions: (a) do you have as much pep as you had last year (*no/yes*); (b) as you get older, are things better or worse than you thought (*better/worse*); (c) are you as happy now as when you were younger (*no/yes*); (d) do you feel that as you get older you are less useful (*no/yes*); and (e) do things keep getting worse as you get older (*no/yes*). Internal reliability was middling (Cronbach’s alpha = 0.65), leading us to explore correlations between items. The item “I am as happy now as I was when I was younger” was the only item that did not significantly correlate with all other items, and there were 24 women (13%) for whom this question was discordant from the other four consistent responses. When this item was removed, it did not substantially alter the overall distribution of responses or change correlations with other variables, but it did slightly increase internal reliability (0.67) despite the lower number of questions. As such, the four-item scale was used in final analyses, as it was determined the fifth item may not have been capturing the same underlying construct. Participants received one point for each positive attitude, with higher scores indicating a more positive ATA and lower scores indicating a more negative ATA. The ATA measure, which had an original possible and actual range of 0–4, was scaled linearly to a 0–100 scale using min-max normalization for ease of interpretation alongside other normalized scales.

### Physical Functioning and Pain

At baseline and at follow-up, the modified HIV Medical Outcome Studies measure assessed physical functioning and pain [42]. For physical functioning, participants were asked six questions on how often their health limited them in completing activities, such as walking uphill or climbing stairs, walking one block, lifting heavy objects, and other activities of daily living. Response options included a three-item Likert scale from *limited a lot* to *not limited at all*. A summary score was computed as the sum of items, re-scaled to 0–100 per standard scoring instructions, where a higher score indicates better physical functioning, and a score of 100 indicates no limitations to physical functioning (Cronbach’s alpha = 0.88).

Pain was measured using the question “how much bodily pain have you generally had during the past 4 weeks,” which ranged from *none* to *very severe*. This was also re-scaled to 0–100, where higher scores indicate greater pain.

### HIV and Clinical Factors

Participants self-reported current antiretroviral use, but antiretroviral use was not included as a covariate as almost all participants with HIV were taking antiretrovirals. At baseline, participants indicated if they had been hospitalized for a medical illness in the past year.

### Depression

Baseline depression was measured using the 20-item Center for Epidemiological Studies Depression Scale (CES-D), which results in a score from 0 to 60 (Cronbach’s alpha = 0.89), with higher scores indicating greater depression symptom severity [43]. Depression scores were dichotomized based on validated CES-D cut-offs for elevated depression symptoms (> or = 16).

### Analysis

**Fig. 1:**
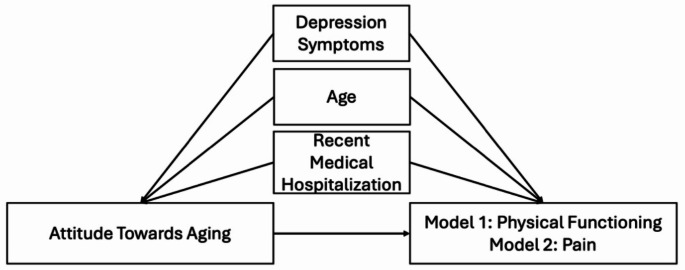
Conceptual model wherein attitudes towards aging are hypothesized to be associated with physical functioning and pain after controlling for the confounding effects of depression symptom severity, age, and hospitalization for medical illness in the past year

Pain and limitations to physical functioning were selected as health-related quality of life outcomes relevant to WWH, who often experience under-treated chronic pain [44, 45]. Covariates (Fig. 1) included biopsychosocial factors potentially related to pain and physical functioning that could also influence ATA, including age, elevated depression symptoms, and past-year hospitalization for a medical illness [13, 24, 28, 31, 46–49]. Depression was included as a covariate because ATA may be a reflection of overall negative attitudes in depression, and there is ample research connecting depression with ATA and physical health [24, 28, 31, 46, 48, 50, 51]. As shown in Fig. 1, depression was hypothesized to serve as a confounding variable, because we theorized that depression in the reproductive years would be a more global cognitive-affective influence that could potentially impact both ATA and pain/physical functioning, in contrast to among older adults, where ATA may take on greater prominence in promoting depressive symptoms. Recent hospitalization for a medical illness was included as a proxy for recent severe illness, because this could impact physical outcomes; in addition, ATA has been associated with hospitalization among older adults [52], and ATA is less positive among older and younger individuals with chronic illness compared to those without [49, 53].

The full ATA questionnaire was only completed by participants at the baseline visit, but self-reported physical functioning and pain questionnaires were completed at the baseline and follow-up visits. For this reason, we first examined the cross-sectional association of baseline ATA with baseline physical functioning and pain in the larger sample. Second, we examined the association of ATA at baseline with follow-up physical functioning and pain in the smaller sample of women with data from two visits.

Descriptive statistics and plots were examined to characterize the sample, identify outliers, assess missing data, and examine distributional assumptions. Differences in mean ATA and individual items from the ATA scale were compared between WWH and WWOH using *t* tests and chi-square tests of independence or Fisher’s exact tests.

The sample was then restricted to WWH. Cross-sectional baseline analyses were conducted first to leverage the larger baseline sample, followed by longitudinal mixed-model regressions with the sub-sample of WWH with two visits. Mixed-effects models included physical functioning and pain at baseline and follow-up as outcomes and allowed intercepts to vary across participants. This mixed-effects approach was selected due to the presence of individual-level variability and to allow for the estimation of the proportion of variance in the outcomes attributable to within-subjects effects.

First, Pearson correlations were examined between all numeric variables at baseline. Linear regressions examined the cross-sectional association of baseline ATA with baseline physical functioning and pain in simple regressions and in multiple regressions controlling for age, elevated depression, and hospitalization for a medical illness in the past year (Fig. 1). Models were initially fit using ordinary least squares regression; however, some models showed heteroscedasticity. In these cases, we used two separate methods for accounting for heteroscedasticity. First, regressions were fit using weighted least squares estimation, where the weights were defined as the reciprocal of the squared fitted values from the regression of the absolute residuals, which creates weights that are inversely proportional to the estimated variance, meaning less extreme data points were given more weight. Second, the *sandwich* package in R was used to obtain robust standard error estimators (i.e., heteroscedasticity-consistent standard errors) from the original linear regression models, which were then used for hypothesis testing [54, 55]. Given the results of sandwich estimation were similar to weighted least squares, the weighted least squares results are presented in full.

In mixed-effects models, baseline ATA, visit (follow-up vs. baseline), and visit*baseline ATA were examined as fixed effects. Covariate-adjusted models then added elevated depressive symptoms as a fixed effect. Additional covariates (age, hospitalization) were not included in the mixed-effect models because they were not significant in prior models, and model parsimony was prioritized given the smaller sample size for longitudinal data paired with the need to include interaction variables. Missing data were minimal and handled using pairwise or listwise deletion, which resulted in the dropping of one individual. Two-tailed alpha was set to 0.05.

Analyses were completed using open-source code in R Version 4.3.1 [56], including the analytic packages *corrplot* [57], *performance* [58], *sandwich* [54, 55], and *lme4* [59].

## Results

### Participants

A total of 519 women were enrolled in STAR at the time of analysis, with 324 excluded due to enrolling after the ATA measure was discontinued and 13 excluded due to pregnancy. This resulted in a final sample of 182 at baseline, including 143 WWH and 39 WWOH (Table 1). Of the 143 WWH, 94 completed the follow-up visit, and 93 had complete data and were included in the follow-up analysis. At baseline, all model variables were similar for those with and without a follow-up visit (*ns*). The average age of participants was 37 (range = 19–45), with 87% of participants being at least 30 years-of-age. About 86% of participants were non-Latina/non-Hispanic Black. Among WWH, all but three individuals were taking ART. There were no significant differences in sociodemographic characteristics or other key variables in Table 1 by HIV status (*ns*).

**Table 1:** Baseline sociodemographic characteristics and key variables by HIV status

Characteristic	Living with HIV (*N* = 143)	Living without HIV (*N* = 39)	Overall (*N* = 182)
	*M* (± *SD*) or *n* (*column* %)		
Age	38 (± 6)	37 (± 6)	37 (± 6)
Age (Categorical)			
18–20	1 (1)	0 (0)	1 (1)
21–25	5 (3)	3 (8)	8 (4)
26–30	16 (11)	5 (13)	21 (12)
31–35	29 (20)	6 (15)	35 (19)
36–40	44 (31)	14 (36)	58 (32)
40–45	48 (34)	11 (28)	59 (32)
Education			
Did not complete high school	40 (28)	10 (26)	50 (27)
Completed high school/GED	48 (34)	14 (36)	62 (34)
Some college or associates degree	38 (27)	11 (28)	49 (27)
Completed four-year college degree or beyond	16 (11)	4 (10)	20 (11)
Difficulty Paying for Basic Needs^1^			
Not very hard	75 (52)	21 (54)	96 (53)
Somewhat hard	45 (31)	12 (31)	57 (31)
Very hard	21 (15)	6 (15)	27 (15)
Race and Ethnicity			
Black, Non-Latina or Hispanic	124 (87)	32 (82)	156 (86)
White, Non-Latina or Hispanic	4 (3)	3 (8)	7 (4)
White, Latina or Hispanic	5 (3)	1 (3)	6 (3)
Multi-Racial or Other, Non-Latina or Hispanic	4 (3)	2 (5)	6 (3)
Multi-Racial or Other, Latina or Hispanic	4 (2)	1 (3)	5 (3)
American Indian/Alaskan Native, Non-Latina or Hispanic	1 (1)	0 (0)	1 (1)
Black, Latina or Hispanic	1 (1)	0 (0)	1 (1)
Taking Antiretroviral Medications	139 (97)	NA	NA
Physical Functioning (0 = worst, 100 = best)	82 (± 25)	86 (± 22)	83 (± 24)
Has Any Limitations to Physical Functioning	80 (56)	18 (46)	98 (54)
Pain (0 = least severe, 100 = most severe)	40 (± 28)	28 (± 28)	33 (± 28)
Has Moderate-to-Very-Severe Pain	34 (28)	8 (21)	48 (26)
Has Elevated Depression Symptoms	58 (41)	10 (26)	68 (37)
Hospitalized in Past Year for Medical Illness^2^	20 (14)	5 (13)	25 (14)

Values are rounded to nearest whole number. ^1^*n* missing = 2. ^2^*n* missing = 1

### Attitudes Towards Aging

As noted in Table 2, having a negative attitude towards aging was common: 32% of women felt things get worse as they get older, 40% did not have as much pep as last year, 13% felt less useful as they got older, 19% felt things as they get older are worse than they thought, and 34% felt they are not as happy now as when they were younger, which was the item removed from the final scale. Excluding the final item, having at least one or more negative attitude about aging was reported by 57% of WWH and 58% of WWOH. Continuous ATA scores ranged from 0 to 100, with a mean of 74 (*SD* = 30).

**Table 2:** Attitudes towards own aging by HIV status

	Living with HIV (*n* = 143)	Living without HIV (*n* = 39)	Overall (*N* = 182)	Statistic	*P*
	*M* (± *SD*)			*t* (57)	
Attitudes Towards Own Aging Summary Score (0–100)	74 (± 30)	73 (± 32)	74 (± 30)	−0.21	0.831
Individual Items	*n* (*column %*)			*X*^2^ (1)	
Things keep getting worse as you get older: *Yes*	47 (33)	12 (31)	59 (32)	0.06	0.804
Do you have as much pep as you had last year: *No*	59 (41)	13 (33)	72 (40)	0.80	0.370
Do you feel that as you get older you are less useful: *Yes*	18 (13)	5 (13)	23 (13)	NA^1^	1
As you get older, are things better or worse than you thought: *Worse*	23 (16)	12 (31)	35 (19)	4.25	0.039
Are you as happy now as you were when you were younger: *No*	50 (35)	11 (28)	61 (34)	0.63	0.428

For attitudes towards own aging, 0 = Most Negative and 100 = Most Positive

The attitude towards own aging summary score was compared across HIV status using a two-sample *t* test

^1^Individual item frequencies were compared across HIV status using chi-square tests of independence, except for one case in which Fisher’s Exact Test was used due to low expected frequencies

In summary, results suggest only the question “as you get older, are things better or worse than you thought” differs between women living with and without HIV

When comparing by HIV status, there were no significant differences for the overall ATA score, with a mean of 73 out of 100 among WWH and 74 out of 100 among WWOH, (*t*(57) = − 0.21, *p* = 0.831). The only difference for individual items was for the question “as you get older, are things *better* or *worse* than you thought?” for which the response of “*worse*” was reported by a greater proportion of WWOH (31%) than WWH (16%), *X*^*2*^(1) = 4.25, *p* = 0.039.

The sample was then restricted to WWH, and ATA was examined in association with proposed covariates. WWH with elevated depressive symptoms had a significantly less positive ATA (Mean = 61) compared to WWH without elevated depressive symptoms (Mean = 83), *t*(87) = 4.1, *p* < 0.001. In Pearson correlations, per Cohen’s [60] correlation classification scale, there was a large negative correlation between ATA and continuous depression scores (*r* = -0.51, *p* < 0.001). As ATA became more positive, depression symptom severity decreased. Neither age nor being hospitalized for a medical illness was associated with ATA (*ns*).

### ATA and Physical Functioning

#### Baseline Analyses

At baseline, 56% (*n* = 80) of WWH reported one or more limitations regarding their physical functioning, with an average score of 82 of 100 (*SD* = 25), where 100 indicates the best physical functioning (Table 1). Pearson correlations showed a moderate positive correlation between ATA and baseline physical functioning (*r* = 0.36, *p* < 0.001), such that more positive ATA was associated with better physical functioning. There was a smaller negative correlation between continuous depression scores and physical functioning (*r* = − 0.26, *p* = 0.002), such that more severe depression severity was associated with worse physical functioning. Age was not correlated with physical functioning.

As shown in Table 3, the weighted least squares model where ATA was used to predict physical functioning at baseline showed good fit, with ATA accounting for about 7% of the variance in baseline physical functioning (*F*(1,141) = 11.43, *p* = 0.001). A one-point increase in ATA, which represented a more positive attitude, was associated with an estimated 0.28 point (95% CI [0.12, 0.44], *p* = 0.001) increase in physical functioning. Using sandwich estimation to account for heteroscedasticity resulted in similar beta estimates (beta = 0.31) and a slightly higher adjusted R-squared (12%) than the weighted least squares model. The multivariable model controlling for age, depression, and recent hospitalization was also significant overall, with model predictors accounting for 12% of the variance in baseline physical functioning (*F*(4,137) = 5.714, *p* < 0.001). All else held constant, ATA (beta = 0.24, 95% CI [0.08, 0.40], *p* = 0.004) and having elevated depression symptoms (beta = − 11.25, 95% CI [− 19.48, − 3.02], *p* = 0.008) contributed to the model’s prediction of baseline physical functioning above-and-beyond other variables. Age and hospitalization were not significantly associated with physical functioning, all else held constant.

**Table 3:** Cross-Sectional regressions examining baseline attitudes towards own aging (ATA) in relationship to baseline physical functioning and pain among 143 women living with HIV across the Southern united States

	Baseline Physical Functioning		Baseline Pain	
	Simple Regression	Multiple Regression^1^	Simple Regression	Multiple Regression^1^
	beta [95% CI]			
(Intercept)	60.89*** [46.11, 75.67]	70.28*** [46.02, 94.53]	45.69*** [33.54, 57.84]	4.73 [−27.12, 36.59]
ATA	0.28*** [0.12, 0.44]	0.24** [0.08, 0.40]	−0.16* [−0.31, −0.01]	−0.07 [−0.22, 0.09]
*Adj. R-Squared*	0.07	0.12	0.02	0.10
*P*	0.00	0.00	0.04	0.00

^1^Multiple regressions controlled for age, depression (elevated vs. normal), and any hospitalization for a medical illness in the past year

***p < 0.001; **p < 0.01; *p < 0.05

Higher scores for continuous variables indicate more positive attitudes towards own aging (ATA), better physical functioning, and more severe pain

In summary, results suggest ATA are significantly associated with physical functioning in a simple regression and after controlling for covariates at baseline

ATA are only weakly associated with pain in a simple regression at baseline, but not after accounting for the effect of covariates

#### Longitudinal Analyses

**Fig. 2:**
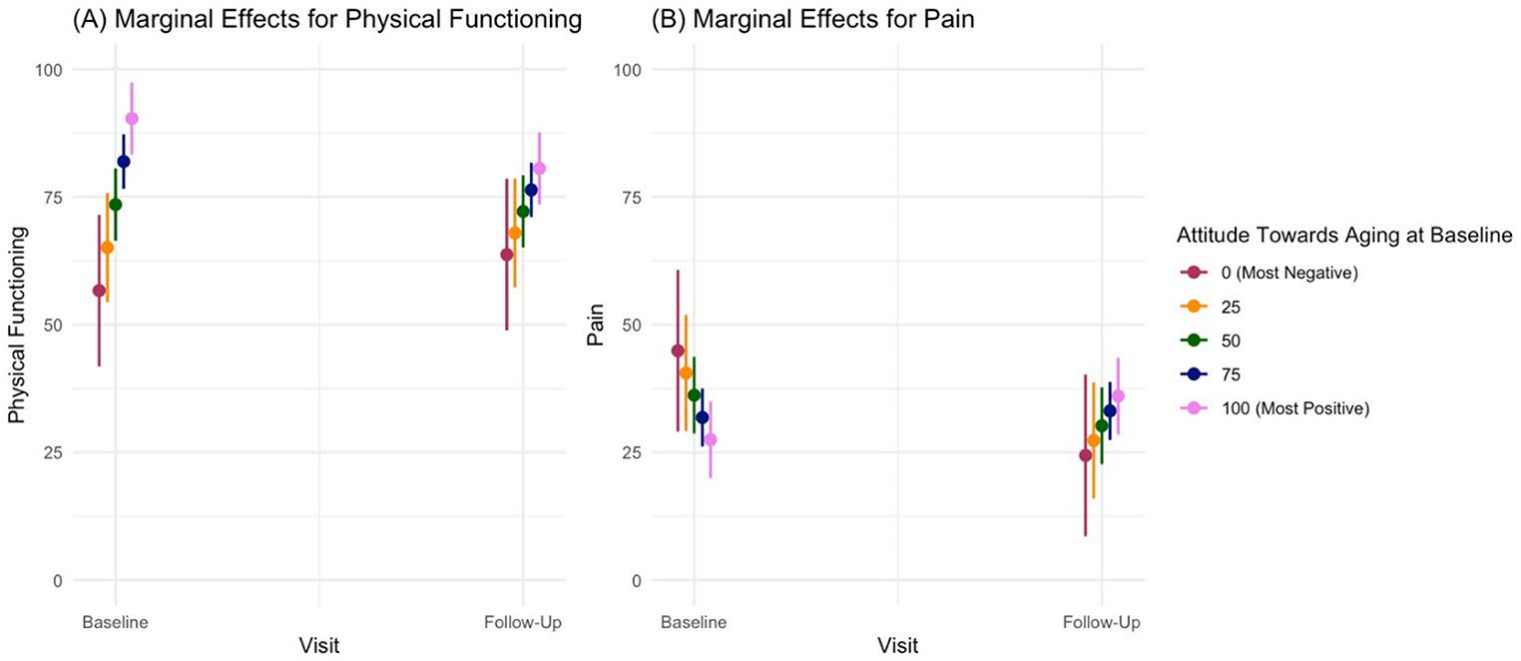
Interaction plots from mixed-effects models fit using data from 93 women living with HIV showing the estimated marginal effects of the interaction between visit (baseline or 6-month follow-up) and baseline attitudes towards aging (ATA), measured as a 0 (most negative) to 100 (most positive) scale, in relationship to the outcomes of **A** physical functioning, which was measured as a 0 (least limitations to physical functioning) to 100 (most limitations to physical functioning) scale and **B** pain, which was measured as a 0 (least severe) to 100 (most severe) scale. Lines represent 95% confidence intervals. For **A**, the interaction was not significant (*p* = 0.09), whereas for **B**, the interaction was significant (*p* = 0.01). The interaction plots indicate that more positive baseline ATA were associated with better physical functioning across visits, although this effect may be slightly attenuated at follow-up in the current sample, where estimated physical functioning showed some regression towards the mean. More positive ATA were associated with less severe pain at baseline, but not at follow-up, as estimates for pain at follow-up across values of baseline ATA had overlapping confidence intervals

For the mixed-effects models, we examined visit (baseline vs. follow-up), baseline ATA, and baseline ATA*visit as fixed effects alongside subject as a random effect (Table 4). Although marginal effects plots show a slight attenuation in the strength of the association between baseline ATA and physical functioning at follow-up compared to baseline, the interaction was non-significant (*p* = 0.09) (Fig. 2). It was estimated that a one-point higher ATA at baseline was associated with a 0.34 (95% CI [0.15, 0.52], *p* < 0.001) point improvement in physical functioning across visits. About 45% of the variance in physical functioning was attributable to within-subject effects.

**Table 4:** Mixed-Effects models examining association of baseline attitudes towards own aging (ATA) in relation to physical functioning and pain measured at baseline and Follow-Up among 93 women living with HIV

	Physical Functioning		Pain	
	beta [95% CI]			
Fixed Effects	Just ATA	Controlling for Depression	Just ATA	Controlling for Depression
(Intercept)	56.66** [41.91, 71.40]	62.96** [47.17, 78.75]	44.89** [29.18, 60.61]	37.68** [21.01, 54.34]
Visit: Follow-Up	7.06 [− 8.41, 22.52]	7.06 [− 8.41, 22.52]	− 20.48* [− 38.60, − 2.37]	− 20.48* [− 38.60, − 2.37]
Baseline ATA	0.34** [0.15, 0.52]	0.30** [0.12, 0.49]	− 0.17 [− 0.37, 0.02]	− 0.13 [− 0.33, 0.06]
Visit*Baseline ATA	− 0.17 [− 0.36, 0.02]	− 0.17 [− 0.36, 0.02]	0.29* [0.06, 0.52]	0.29* [0.06, 0.52]
*AIC/BIC*	1726/1745	1719/1741	1759/1779	1751/1773
*Pseudo R*^*2*^*(fixed)*	0.09	0.12	0.02	0.06
*Pseudo R*^*2*^*(total)*	0.50	0.50	0.35	0.36
ICC for Subject	0.45	0.44	0.34	0.32

***p < 0.001; **p < 0.01; *p < 0.05

P values were computed using Satterthwaite degrees of freedom

Higher scores for continuous variables indicate more positive attitudes towards own aging (ATA), better physical functioning, and more severe pain

Reference category for visit is “Baseline”

Results indicate that ATA at baseline were associated with physical functioning across visits when modelled with just ATA and visit, as well as when controlling for depression symptoms

In the models for pain, there was a significant interaction between visit and baseline ATA, which when probed suggested baseline ATA were associated with baseline pain, but not follow-up pain

Adding baseline depression to the model slightly improved model fit, and ATA remained significantly associated with physical functioning across visits (beta = 0.30, 95% CI [0.12, 0.49]), *p* = 0.01), depression held constant. Having elevated depression was also associated with worse physical functioning across visits, all else held constant (beta = − 9.65, 95% CI [−18.97, − 0.34], *p* = 0.05).

In terms of clinically relevant categories, among WWH with two visits, 31% (*n =* 29) had no limitations to physical functioning at either visit, 9% (*n =* 8) improved from having at least one limitation to none, 14% (*n =* 13) worsened from no limitations to at least one limitation, and 46% (*n =* 43) had limitations at both visits. Mean baseline ATA and the proportion of WWH with elevated depressive symptoms at baseline were compared across these categories. There were no statistically significant differences across groups, although descriptively, baseline ATA was most positive among WWH with no physical limitations at either visit (Mean ATA = 84) compared to WWH with limitations at just baseline (Mean ATA = 69), at just follow-up (Mean ATA = 67), or at both visits (Mean ATA = 73).

### ATA and Pain

#### Baseline Analyses

At baseline, 71% (*n* = 102) of WWH reported recent pain, with 41% (*n* = 62) having very mild/mild pain, 17% (*n* = 25) having moderate pain, and 10% (*n* = 15) having severe/very severe pain. The average pain score was 34 of 100 (*SD =* 28), where higher scores indicate worse pain. Pearson correlations at baseline showed a small negative correlation between ATA and pain (*r =* − 0.17, *p* = 0.04), such that more positive ATA was associated with decreased pain severity. There was a positive correlation between continuous depression scores and pain (*r* = 0.26, *p* = 0.002), such that increased depression symptom severity was associated with worse pain severity. Age did not correlate with pain.

In the cross-sectional baseline model, ATA accounted for a small proportion (2%) of the variance in baseline pain (*F*(1,141) = 4.31, *p* = 0.04). For each point increase in ATA, estimated pain severity decreased − 0.16 points (95% CI [− 0.01, − 0.31], *p* = 0.04). When covariates were added to the model, the normality of residuals was improved, and the adjusted model accounted for 10% of the variance in baseline pain *F*(4,137) = 4.84, *p* = 0.001. ATA no longer predicted pain (beta = − 0.07, 95% CI [− 0.22, 0.09], *p* = 0.405) above-and-beyond the associations of other covariates in the model, but there was a significant association between elevated depressive symptoms and worse pain (beta = 15.92, 95% CI [6.35, 25.48], *p* = 0.001), all else held constant.

#### Longitudinal Models

In the mixed-effects model including visit, baseline ATA, and visit*baseline ATA, within-person effects accounted for 34% of the variation in pain. Although pain severity decreased across time on average (beta = −20.48, 95% CI [−38.6, −2.37], *p* = 0.03), there was a significant interaction between visit and ATA (beta = 0.29, 95% CI [0.06, 0.52], *p* < 0.01). An examination of interaction plots (Fig. 2) for predicted values of pain suggests baseline ATA was positively associated with decreased pain at baseline, but this effect was diminished at follow-up. When depression was added to the model, having elevated depressive symptoms at baseline was associated with more severe pain across visits (beta = 11.05, 95% CI[1.58, 20.52], *p* = 0.02), and other results were unchanged.

In terms of clinically relevant categories, among WWH with two visits, 55% (*n* = 51) had no/mild pain at both visits, 15% (*n* = 14) had pain that improved from moderate/severe to no/mild, 18% (*n* = 17) had pain that worsened from no/mild to moderate/severe, and 12% (*n* = 11) had moderate/severe pain at both visits. Baseline ATA was most negative among WWH with moderate/severe pain at both visits (Mean ATA = 61) and more positive among WWH without pain at baseline, including WWH without pain at follow-up (Mean ATA = 77) and WWH with pain at follow-up (Mean ATA = 84), but this did not reach statistical significance. However, the proportion of participants with elevated depression symptoms at baseline was significantly different across groups (*p* = 0.028) in a Fisher’s exact test, with the lowest rates of elevated depression symptoms among WWH with no/mild pain at both visits (25%, *n* = 13) in comparison to moderate/severe pain at both visits (45%, *n* = 5), moderate/severe pain at baseline that improved at follow-up (57%, *n* = 8), and no/mild pain at baseline that worsened at follow-up (59%, *n* = 10).

## Discussion

This study evaluated ATA among reproductive-age women enrolled in a longitudinal cohort study in the southern US. There are clearly unmet needs for improving ATA, health-related quality of life, and mental health among WWH prior to mid-life, with 57% reporting at least one negative attitude towards aging, 28% having moderate-to-severe pain, 56% reporting physical limitations, and 41% having elevated depression symptoms. WWOH and WWH had similar attitudes towards aging, suggesting efforts to improve conceptualizations of aging are warranted in both populations, although co-morbidities such as pain, depression, and limitations to physical functioning were most common among WWH. Given the paucity of research on ATA and health outcomes among women of reproductive age, results from the current study suggest examining ATA’s associations with health outcomes is warranted prior to midlife.

It was hypothesized that more positive ATA at baseline would be associated with better physical functioning at baseline and at a 6-month follow-up visit, and that these associations would remain significant after adjusting for theoretically selected covariates. In line with our hypotheses, among WWH, ATA were related to physical functioning at baseline, including in simple regressions without covariates in the model and after controlling for age, depression, and recent hospitalization. ATA were also related to physical functioning across visits in mixed-effects models, including without covariates in the model and when controlling for depression. Although non-significant, an examination of clinically relevant change variables suggests ATA at baseline was most positive among WWH with no current or future limitations to physical functioning in the current sample, although this may not be generalizable to the population. Taken together, these findings highlight the potential for a temporal relationship wherein ATA may predict physical functioning, but this requires evaluation over a longer period. To elucidate the mechanisms connecting these factors among WWH, experimental research is needed to test if cognitive-behavioral interventions to improve ATA and depression can enhance physical functioning, and - in the opposite direction - if pharmacological or physical therapy interventions to improve physical functioning can improve depression and ATA.

It was also hypothesized that more positive ATA would be associated with better pain at baseline and at follow-up. In contrast to our hypothesis, ATA showed a weak negative association with pain at baseline that was no longer significant after accounting for the relationship between depression and pain. In mixed-effects models, more positive baseline ATA was correlated with lower pain at baseline but not at follow-up, while elevated depression was associated with higher pain across visits. Thus, in the current study, ATA was not strongly associated with pain, and any association may have been due to correlations between ATA and depression. Our findings are well-aligned with prior research that has established that depression and pain are common co-morbid conditions [47, 61].

Although depression was not the primary focus of this study, depression was associated with ATA, pain, and physical functioning. While findings on depression are expected due to a large body of research on the health impacts of depression [28, 46, 48], the prominence of depression across models emphasizes the ongoing relevance of depression among WWH and highlights the difficulty of addressing cognitive factors such as ATA without also considering more global depressive symptoms. Altogether, this study’s findings are aligned with prior research from the general population of older adults without HIV [17] and from studies done in men with HIV [32] showing associations between ATA and depression. This study is the first to show the association of ATA with concurrent depressive symptoms and longitudinal physical functioning among reproductive-age WWH and supports the ongoing need for more effective treatments for co-morbid depression and pain in WWH.

### Implications for Research and Practice

Despite the observational nature of our findings, it is notable that directly modifiable cognitive and psychological factors—ATA and depressive symptoms—but not age or hospitalization, were associated with physical functioning and pain. This finding is aligned with psychological theories of aging, including Flood’s theory of successful aging, which emphasizes the importance of psychological coping with physical limitations in aging, as well as cognitive-behavioral and stress-coping theories, which emphasize the role of cognitions and behaviors in promoting health-related quality of life in the context of chronic disease [5, 62, 63]. It may therefore be possible to improve pain and physical functioning through a combination of cognitive-behavioral, pharmacological, and physiological interventions [62, 64, 65]. Cognitive-behavioral interventions have been tested to reduce pain and improve health-related quality of life among people living with HIV and people without HIV; these interventions show mostly promising results, although some studies showed moderate-to-high drop-out rates, and implementation in HIV and primary care clinics has not been widespread [62, 65–69]. Cognitive-behavioral interventions have also been studied for efficacy in promoting HIV-specific behavior change (e.g., sexual risk behavior, HIV knowledge) in older adults living with HIV, suggesting cognitive-behavioral interventions could be tailored to meet needs of WWH across the lifespan [70]. The current study and prior research on the link between depression and pain highlight the need to expand the availability of efficacious interventions tailored for WWH with co-morbid pain and depression [65, 71–73]. Self-compassion has been identified as a potentially modifiable factor associated with ATA among women in midlife that could be added to ATA interventions [74]. Research is also needed to determine if negative attitudes specific to aging require unique approaches in comparison to negative attitudes resulting from depressive or other mental health symptoms.

Research is needed to assess if adding content to address ATA alongside depression and chronic pain can further improve health-related quality of life or increase intervention engagement and retention among women. Research suggests acceptance-based interventions to enhance positive aging among midlife and older adults are feasible and acceptable [75, 76], but findings about the ability for acceptance-based interventions to decrease internalized ageism among adults is mixed [77]. One study found that an acceptance-coping and mindfulness-based intervention decreased ageism among college students, but increased internalized ageism among older adults [77]. The AgingPLUS Program is an intervention focused on reducing negative ATA, increasing self-efficacy, and improving goal setting to increase physical activity among older adults [33]. In a trial comparing AgingPLUS to a health education condition, individuals in AgingPLUS showed greater improvements in ATA, self-efficacy, and behavioral intentions than in the control group, but both groups had similar improvements in physical activity [33, 78, 79]. Given these promising but conflicting results, studies on the effectiveness of cognitive-behavioral therapies to improve depressed mood and ATA among reproductive-age women are needed.

Lastly, the findings of our study may also be interpreted through a social/environmental framework such as the Risks of Ageism Model, which proposes that the association of ATA with aging outcomes occurs due to stereotype embodiment (i.e., internalization), stereotype threat (i.e., perceived risk of conforming to stereotypes, which can cause stress), and age discrimination [80]. Findings from an observational study of Italian adults 18–64 suggests that better quality and amount of contact with older adults is linked to more positive ATA across age groups, including among younger people [81]. Consequently, research should investigate the beneficial effects of increasing reproductive-age women’s contacts with successfully aging women and explore strategies to decrease age segregation, which may reduce stereotypes about aging among younger individuals.

### Limitations

This is an observational study, so uncontrolled confounding cannot be ruled out. Although our conceptual model (Fig. 1) represents hypothesized causal pathways, our findings reflect associations rather than definitive causal relationships. The possibility that ATA influences depressive symptoms, or that an unmeasured variable impacts ATA, depression, and health-related quality of life cannot be ruled out. Experimental research to improve ATA is needed to confirm causal pathways. Most women were Non-Latino Black, and all were living in the southern US, which reflects the demographics of HIV in the US but prevents a comparison of findings by race or ethnicity and region. Cultural factors may impact ATA, so this is an area of importance for further investigation. To understand whether the ATA measure should be adapted for WWH, research is needed to examine why one item on the ATA measure (“are you as happy now as when you were younger”) was not correlated with the other items. It is possible that this item is capturing the higher prevalence of negative childhood experiences among WWH [82]. Future research should confirm if findings are correlated with objective measures of physical functioning. Lastly, since our study focused on a reproductive-age cohort, research is needed to assess ATA over time and confirm if ATA during the reproductive years predicts successful aging in mid-life and older age.

### Generalizability

In this multi-site study, ATA did not differ by HIV status or age, which is consistent with prior research among men that did not find differences in ATA across HIV status [32]. Rates of moderate-to-severe pain among WWH in the current sample were similar to rates of chronic pain among WWH reported globally [45]. Together, this suggests findings may be reasonably generalizable across women 18–45 years-of-age with and without HIV living in southern US cities. Based on prior research showing rural/urban and age-related differences in ATA, additional studies may be needed to assess whether findings are generalizable to men and women of different ages residing in other settings [83, 84].

## Conclusions

WWH reported negative ATA and high levels of depressive symptoms, pain, and limitations to physical functioning starting prior to mid-life. Among WWH, depression and self-perceptions of aging, rather than age, were correlated with each other and with pain (for depression) and physical functioning (for depression and ATA). As women with HIV are experiencing premature aging and demonstrate negative ATA in concert with depressive symptoms, research is needed to facilitate healthy aging attitudes and experiences and improve mental health among WWH starting in the reproductive years. Similarly, many WWOH indicated holding negative ATA, indicating a broad need for programs to improve women’s self-perceptions of aging in the southern US.

## Data Availability

Raw data from this study are maintained at the STAR Data Coordinating Center. To maintain participant confidentiality, raw data and reproducible analytic code are available from STAR upon the approval of an investigator concept sheet (https://statepi.jhsph.edu/star/work-with-us/).
